# Supplementary material for: Polymorphisms in Pfkelch13 domains before and after the introduction of artemisinin-based combination therapy in Southwest Nigeria
Source: PLoS One. 2025 Mar 31;20(3):e0316479. doi: 10.1371/journal.pone.0316479 (PMC11957316; doi:10.1371/journal.pone.0316479)
Supplement: Supporting information 2 — (ZIP) [file pone.0316479.s002.zip › 041KN2F_PREMIX_Plate_KELCH1_H08.pdf]

Samples: 13437
Bases: 1090
Average spacing: 13

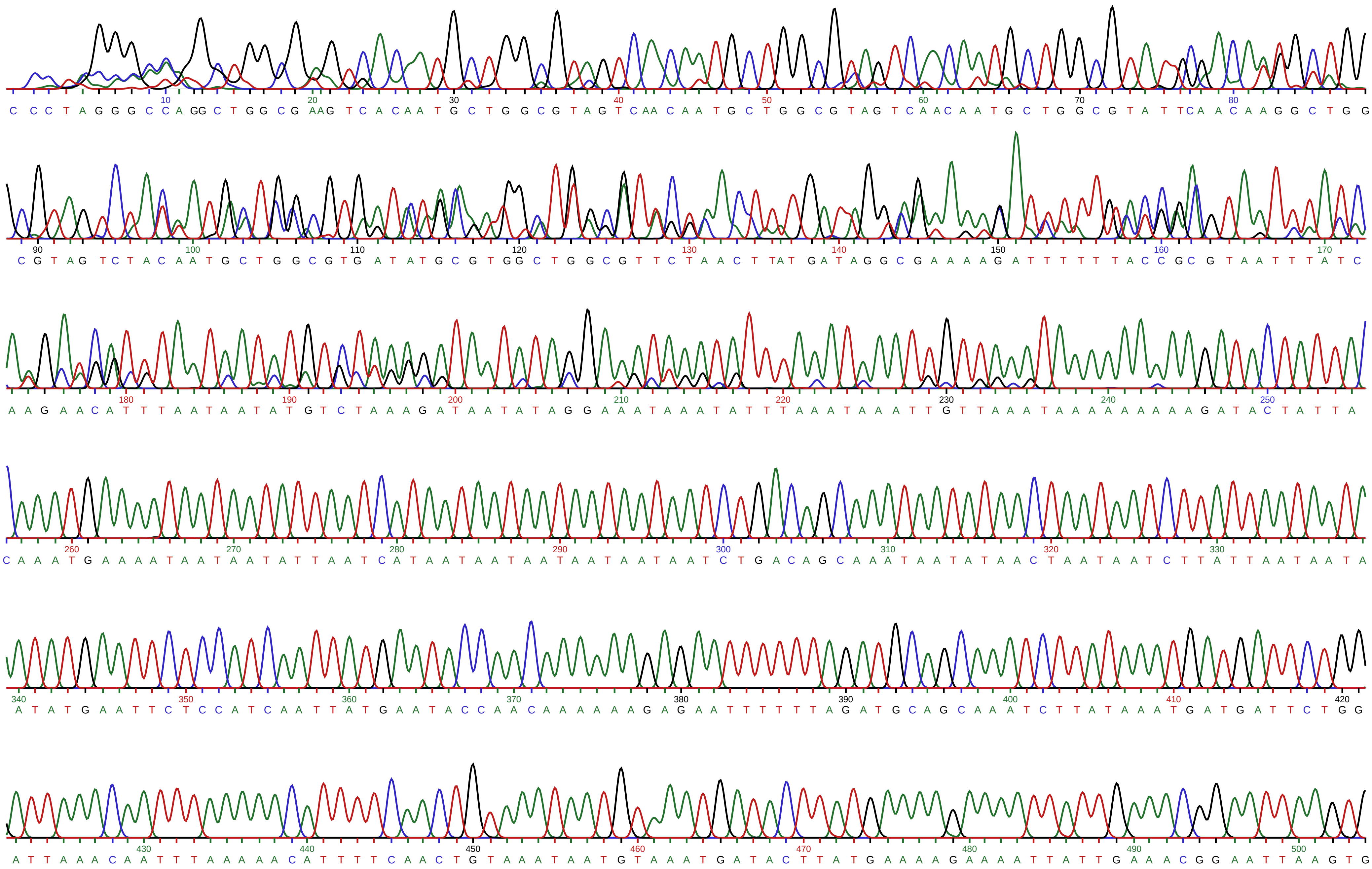

Samples: 13437
Bases: 1090
Average spacing: 13

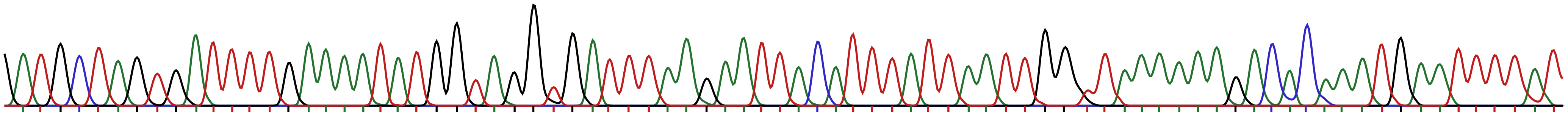

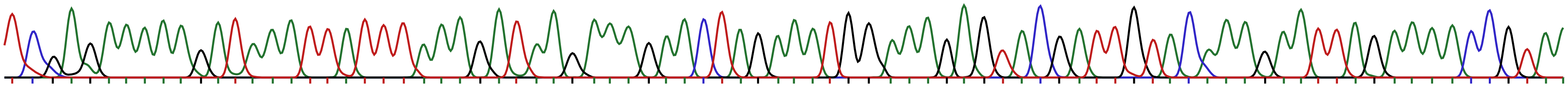

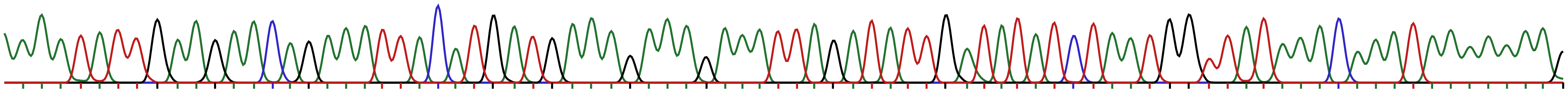

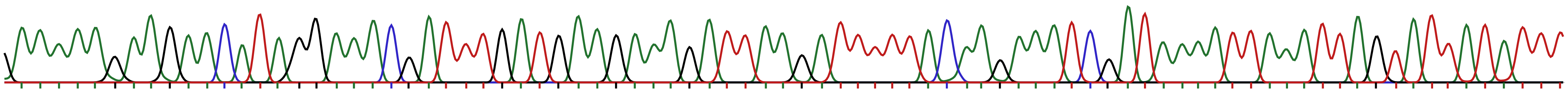

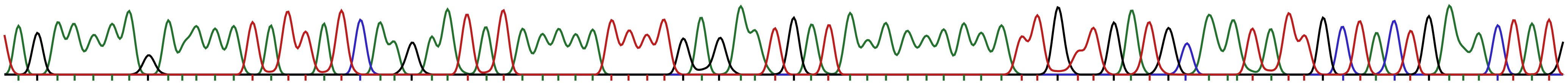

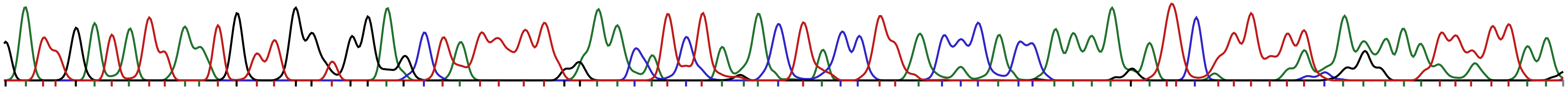

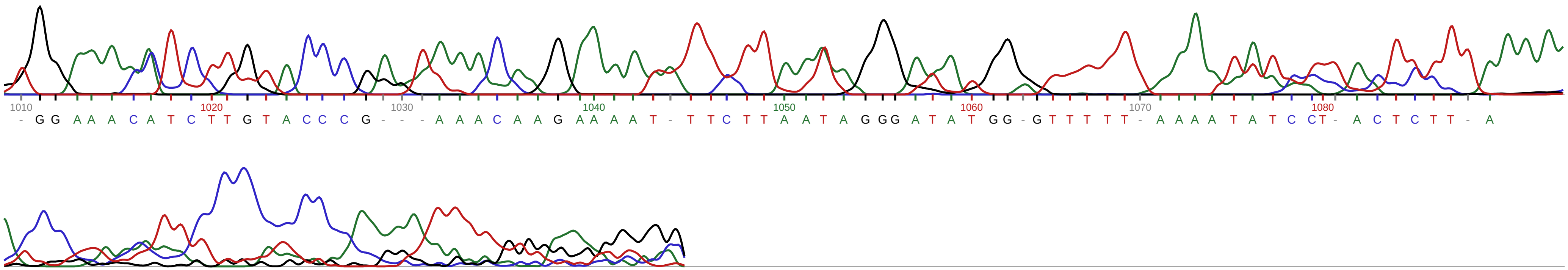
1010 1020 1030 1040 1050 1060 1070 1080
- G G A A A C A T C T T G T A C C C G - - - A A A C A A G A A A A T - T T C T T A A T A G G G A T A T G G - G T T T T T - A A A A T A T C C T - A C T C T T - A
